# Textbook Outcome in Colorectal Surgery for Cancer: An Italian Version

**DOI:** 10.3390/jcm13164687

**Published:** 2024-08-09

**Authors:** Silvia Sofia, Maurizio Degiuli, Gabriele Anania, Gian Luca Baiocchi, Ludovica Baldari, Gianandrea Baldazzi, Francesco Bianco, Felice Borghi, Davide Cavaliere, Claudio Coco, Roberto Coppola, Domenico D’Ugo, Paolo Delrio, Uberto Fumagalli Romario, Mario Guerrieri, Marco Milone, Mario Morino, Andrea Muratore, Giuseppe Navarra, Corrado Pedrazzani, Roberto Persiani, Wanda Petz, Riccardo Rosati, Franco Roviello, Stefano Scabini, Giuseppe Sica, Leonardo Solaini, Antonino Spinelli, Gaya Spolverato, Emanuele Urso, Rossella Reddavid

**Affiliations:** 1 Department of Oncology, Division of Surgical Oncology and Digestive Surgery, San Luigi University Hospital, University of Turin, 10043 Turin, Italy; sofiaasilvia@gmail.com (S.S.); 2 Dipartimento Scienze Mediche, Università di Ferrara, 44121 Ferrara, Italy; g.anania@unife.it; 3 Department of Clinical and Experimental Sciences, University of Brescia, 25123 Brescia, Italy; gianluca.baiocchi@unibs.it; 4 Fondazione IRCCS, Ca’ Granda-Ospedale Maggiore Policlinico di Milano, 20122 Milan, Italy; ludovica.baldari@policlinico.mi.it; 5 Divisione di Chirurgia Generale, Nuovo Ospedale di Legnano-ASST Ovest Milanese, 20025 Legnano, Italy; gbaldazzi@hotmail.com; 6 General Surgery Unit, San Leonardo Hospital, ASL-NA3 Sud, Castellammare di Stabbia-Naples, 80053 Naples, Italy; bianco.bar@tin.it; 7 Oncologic Surgery Unit, Candiolo Cancer Institute, FPO-IRCCS, 10060 Turin, Italy; felice.borghi@ircc.it; 8 General and Oncologic Surgery, Morgagni-Pierantoni Hospital, Ausl Romagna, 47121 Forlì, Italy; cavalied@gmail.com (D.C.); leonardo.solaini2@unibo.it (L.S.); 9 Fondazione Policlinico Universitario A. Gemelli–IRCCS, Chirurgia Generale Presidio Columbus, Università Cattolica del Sacro Cuore, 00168 Rome, Italy; claudio.coco@unicatt.it; 10 Fondazione Policlinico Universitario Campus Bio-Medico, 00128 Rome, Italy; r.coppola@policlinicocampus.it; 11 Fondazione Policlinico Gemelli–IRCCS, AREA di Chirurgia Addominale, 00168 Rome, Italy; domenico.dugo@unicatt.it (D.D.); roberto.persiani@unicatt.it (R.P.); 12 Colorectal Surgical Oncology, Abdominal Oncology Department, Fondazione Giovanni Pascale IRCCS, 80131 Naples, Italy; delrio.paolo@gmail.com; 13 Digestive Surgery, European Institute of Oncology–IRCCS, 20141 Milan, Italy; uberto.fumagalliromario@ieo.it; 14 Department of General Surgery, UNIVPM IRCCS INRCA, 60121 Ancona, Italy; guerrieri.m@libero.it; 15 Department of Clinical Medicine and Surgery, “Federico II” University of Naples, Via Sergio Pansini 5, 80131 Naples, Italy; milone.marco.md@gmail.com; 16 Department of Surgical Sciences, University of Torino, 10124 Torino, Italy; mario.morino@unito.it; 17 Department of General Surgery, E. Agnelli Hospital, 10064 Pinerolo, Italy; amuratore67@gmail.com; 18 Department of Human Pathology of Adult and Evolutive Age, Surgical Oncology Division, “G. Martino” Hospital, University of Messina, 98125 Messina, Italy; gnavarra@unime.it; 19 Unit of General and Hepatobiliary Surgery, Department of Engineering for Innovative Medicine (DIMI), University of Verona Hospital Trust, University of Verona, 37129 Verona, Italy; corrado.pedrazzani@gmail.com; 20 Department of General and Minimally Invasive Surgery, Ospedale San Paolo, University of Milano, 20142 Milano, Italy; wanda.petz@asst-santipaolocarlo.it; 21 IRCCS San Raffaele Scientific Institute and San Raffaele Vita-Salute University, 20132 Milan, Italy; rosati.riccardo@hsr.it; 22 Unit of General Surgery and Surgical Oncology, Department of Medicine, Surgery, and Neurosciences, University of Siena, 53100 Siena, Italy; franco.roviello@gmail.com; 23 Chirurgia Generale ad Indirizzo Oncologico, Ospedale Policlinico San Martino, 16132 Genova, Italy; stefanoscabini@libero.it; 24 Department of Surgery, University Tor Vergata, 00133 Rome, Italy; sigisica@gmail.com; 25 Department of Biomedical Sciences, Humanitas University, 20072 Pieve Emanuele, Italy; antonino.spinelli@hunimed.eu; 26 IRCCS Humanitas Research Hospital, Via Manzoni 56, 20089 Rozzano, Italy; 27 Section of Surgery, Department of Surgical, Oncological, and Gastroenterological Sciences, University of Padova, 35122 Padova, Italy; gaya.spolverato@unipd.it; 28 General Surgery 3, Department of Surgical, Oncological and Gastroenterological Sciences (DiSCOG), University of Padova, 35122 Padova, Italy; edl.urso@unipd.it

**Keywords:** textbook outcome, colorectal cancer, colorectal surgery, composite measure, patient outcome, quality of care

## Abstract

**Background/Objectives**: The textbook outcome (TO) is a composite tool introduced to uniform surgical units and regulate surgical quality and outcomes. A patient is considered TO only if all predetermined items are met. In colorectal surgery, TO represents a new tool that can achieve important results given the prevalence of colorectal cancers. However, at present, there is a lack of uniformity in the TO’s definition. This study utilized the Delphi process to define an Italian version of the TO in colorectal cancer. **Methods**: The survey consisted of two rounds of online questionnaires submitted to an expert panel in colorectal oncological surgery, renowned academic surgeons, who had attended multiple scientific conferences and who were authors of papers on this specific topic. Five main topics with 26 questions were investigated through an online modified Delphi method. Items with almost 75% agreement achieved consensus. **Results**: Twenty-eight Italian experts were selected and participated in the two rounds. The Italian version of the textbook outcome in colorectal surgery was defined as the presence of 90-day postoperative survival, negative margins and at least 12 lymph nodes, a minimally invasive approach, ostomy fashioning if preoperatively planned, postoperative complication < Clavien–Dindo 3b, at least 10 ERAS items, no readmission, proper CHT and RT regimens, complete colonoscopy after or before surgery and Tumor Board Evaluation. **Conclusions**: The textbook outcome in colorectal cancer patients is a quality instrument providing a complete overview of the care of such patients, from diagnosis to treatment. We hereby propose an Italian version of the TO with outcomes chosen by an expert panel.

## 1. Introduction

Worldwide, colorectal cancer (CRC) is ranked as the 3rd most frequently diagnosed cancer in both sexes. Because of its poor prognosis, it is responsible for the second highest cancer-related death rate, accounting for 904,019 deaths/year in 2022 [1,2]. Resection with negative macroscopic and microscopic margins and adequate lymphadenectomy is the mandatory goal of surgery for localized CRC, according to the worldwide guidelines [3,4,5]. However, both colon and rectal resections are invasive procedures with non-negligible postoperative morbidity and mortality rates [6,7,8,9,10].

In 2013, a research group from the Netherlands first introduced the concept of a textbook outcome (TO) applied to colon cancer resections, a multidimensional measure representing an ideal course after surgery [11].

The authors identified six perioperative quality-of-care parameters: hospital survival, radical resection, no reintervention, no ostomy, no adverse outcome and a hospital stay <14 days. The aim of this study was to define positive and negative outlier hospitals based on the average TO. The textbook outcome gives a simple comprehensive summary of hospital performance that is meaningful for patients, providers, insurance companies and healthcare inspectorates.

The TO represents an attempt to define a standardized measure of quality based on multiple perioperative endpoints that contribute to the definition of optimal care of the patient that is defined as “textbook”. In the last decade, the TO has been proposed for different type of surgeries [12,13,14], with potential promising benefits, especially for cancer surgery, where strategies of care are established by a multidisciplinary team.

However, the use of the TO is actually debated because some authors assert that a single parameter is too reductive to describe such a complex process as the postoperative course [15,16,17]. In fact, it can be influenced by numerous factors depending not only on the quality of surgery but also on the comorbidities of the patient, preoperative treatments, and the general conditions of the patient, such as obesity, cachexia and social aspects.

This study aims to establish an Italian expert consensus on the definition of TO in colorectal surgery among expert colorectal surgeons using a modified Delphi method.

## 2. Materials and Methods

This study was designed and conducted according to CREDES guidelines for studies with a Delphi method consensus [18]. This method was chosen for the present study because it allows us to reach agreement on predetermined topics among selected experts in the field when it is not possible to design studies such as clinical trials. It consists of the preliminary selection of a panel composed of Italian leading experts in both colon and rectal surgery and two subsequent rounds to complete the Delphi process.

### 2.1. Selection of Expert Panel

Suitable members of the expert panel were identified among surgeons from high-volume centers. The participating experts should have performed at least 100 colorectal resections in the previous 5 years; otherwise, they should have an H-index of more than 20. Furthermore, the experts should be authors or coauthors of high-impact publications on colorectal surgery; they should have been invited speakers at national or international meetings on oncological colorectal surgery, and they should have previously participated in multicenter international projects in colorectal surgery. Those who satisfied these criteria were considered eligible and leading experts in colorectal surgery.

### 2.2. Delphi Process

The Delphi process is a structured method that adopts a series of questionnaires or ‘rounds’ to collect information. The rounds are repeated until panel consensus is achieved. This method is very useful because a lot of specialists from different locations and fields of expertise can be involved anonymously, thus avoiding domination of the consensus process by one or a few panelists [19]. Three authors (S.S., M.D. and R.R.) conducted literature research to identify all variables that could be included in this TO version. The questionnaire focused on the perioperative assessment of a patient with colon or rectal cancer undergoing surgical treatment.

The survey consisted of twenty-six questions divided into five topics: postoperative survival, oncological radicality, surgical items, postoperative course and perioperative treatments and diagnostics. At the beginning of the study, two different rounds were scheduled for the submission of the questionnaire to the respondents. Each statement involved a response according to a 5-point Likert scale, from strongly disagree (1) to strongly agree (5). The threshold to achieve consensus was set at 75% agreement among the 28 voting experts, for both negative and positive consensuses.

The panelists had to write a comment for each question; then, the comments were used anonymously in subsequent rounds so that the experts could justify their answers, evaluate the percentages of other experts and eventually modify their own answers.

Based on the score obtained from the responses in the first round, statements that did not achieve consensus were discussed again in the next round to examine the topic and evaluate the inclusion or definitive exclusion of the statement from the final version of the Italian TO. Additional rounds were scheduled if consensus was not reached for at least 50% of the statements. The entire process of selecting experts and voting on individual rounds was conducted electronically, thus ensuring an adequate level of anonymity throughout the study. Both rounds 1 and 2 are available in the Supplementary Materials (S1).

### 2.3. Data Management and Statistical Analysis

The study data were collected and managed through the EU Survey portal (version v1.5.2.9); the data were therefore exported into a Microsoft Excel sheet (v 16.77.1). Statistical analysis was performed using the Microsoft Excel sheet (v 16.77.1), and medians and interquartile ranges were calculated for each variable. Items with almost 75% agreement (agree (3), strongly agree (4) and completely agree (5)) met the inclusion criteria, while those responses with more than 75% disagreement (strongly disagree (1) or disagree (2)) were returned to the preceding phases.

The level of agreement was assessed using descriptive measures as median values, while the interquartile range (IQR) was used as a measure of the dispersion of the level of responses. An acceptable grade of variability was considered to be an IQR of less than 2 [20].

## 3. Results

An expert panel of 28 surgeons who had met all pre-established inclusion criteria was selected.

The group comprised experts from all Italian referral centers for surgical colorectal oncology. Table 1 lists the main features of the 28 panelists who participated in the Delphi rounds of the present study. The panel constituted 25 males and 3 females, with a greater provenience from academic hospitals (96.4%). The majority of panelists (89.3%) declared more than 10 years of clinical expertise in colorectal surgery. The overall academic exposure was high, with approximately 40% of experts having completed more than 100 scientific papers and all of them having participated in national and international meetings as invited speakers.

**Figure 1:**
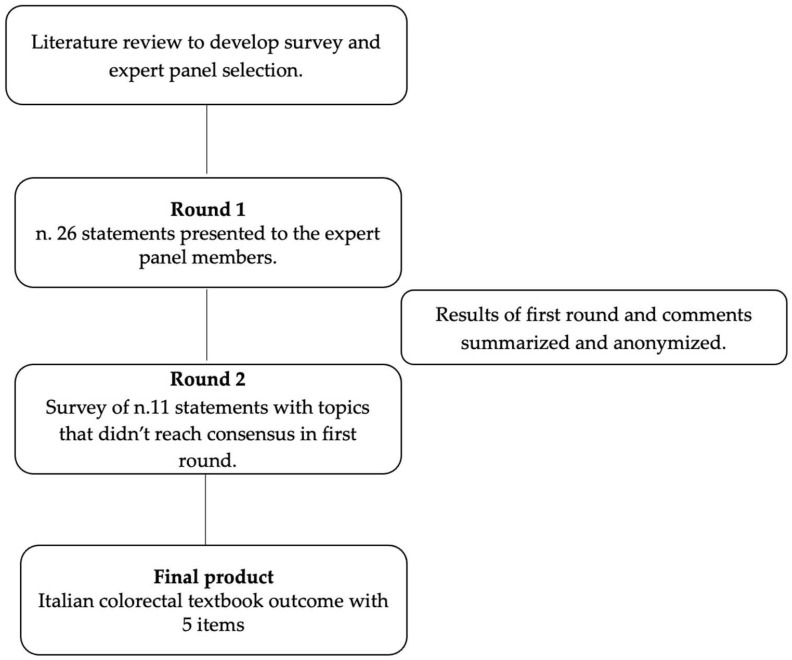
Overview for the Delphi consensus process.

The two Delphi rounds were conducted from July 2022 to September 2023. The response rates were very high for both the first and the second Delphi rounds, with percentages of 100% and 96.4%, respectively. In the first round, agreement was reached for fifteen statements, while eleven statements did not pass the first round and were formulated again in the second round. The Delphi process is detailed in Figure 1. At the conclusion of the two rounds of the Delphi process, consensus was reached among the experts when considering which items should constitute the objective measure of a TO for colorectal surgery and within which time frame the outcomes should be considered.

- Statement 1—Postoperative Survival

The first statement is about patient survival after colorectal surgery. In the first round, the panelists expressed their opinions on which time frame outcomes should be considered. Overall, 75% concurred that the TO should reflect care within 90 days of the colorectal resection.

- Statement 2—Oncological Radicality

The panelists expressed their opinions on the best definition of cancer radicality to be included within the TO. In total, 82.1% of experts agreed to avoid the use of a general definition in favor of a more specific description (statement n. 2.1). Consensus was obtained in the first round to include both the negative histologic margins of the surgical specimen (85.7% response rate) and at least 12 lymph nodes harvested during surgery (78.6%). Finally, margin of at least 5 cm was considered mandatory for colic resections (statement n. 2.7 with 85.7% of agreement in the first round).

- Statement 3—Surgery

The respondents fully agreed on the use of minimally invasive (MI) technique in the absence of anesthesiologic contraindications or clinical reasons.

In the first round, ostomy fashioning for clinical necessity failed to reach agreement (39.3%), while in the second round, it reached consensus (92.6%) with the supplement of “deviation from a regular course only in case of unplanned settings”.

- Statement 4—Postoperative course

The majority of panelists concurred that the TO should include the absence of re-operation (82.1%), postoperative complications (78.6%) and hospital readmission (85.7%) within the first 30 days after surgery. In the second round, these aspects were incorporated into a more generic definition of grade 3b according to the Clavien–Dindo classification (88.9%) [21].

Full agreement among respondents was reached regarding the application of Enhanced Recovery after Surgery protocols (ERAS) [22] in terms of adherence to more than 10 items and only if applicable based on patient’s comorbidities. Postoperative length of stay (LOS) was a hot topic; indeed, neither the first nor the second round reached agreement. Hence, it was removed from the final version of the TO.

- Statement 5—Perioperative treatments and diagnostics

All three items of the last statement reached full agreement among the voters, who considered essential (1) a multidisciplinary evaluation with scheduled tumor board meetings (96.4% agreement in the first round), (2) a complete colonoscopy before or after surgery (75.0%) and (3) an appropriate neoadjuvant and/or adjuvant chemo-radiotherapy treatment scheme.

The rates of agreement and median values with IQRs are reported in Table 2 and Table 3. Final consensus was reached for all five items, (Table 4) and statements that did not pass even the second round were excluded from the final version of the TO.

## 4. Discussion

The textbook outcome is a composite tool introduced to inform surgical units and regulate surgical quality and outcomes. It could positively affect daily clinical practice, for example, by helping patients to select the best surgical center, standardizing techniques, leading to better adherence to guideline indications for the diagnosis and treatment of oncological diseases, and reducing costs through a mechanism of centralizing complex cases in referral centers. In colorectal surgery, the TO represents a new tool that can achieve important results given the prevalence of colorectal cancers.

However, at present, there is a lack of uniformity in the TO’s definition despite the standardization sought by the authors of the TO [23].

The present study aims to provide an Italian expert consensus-based definition of the colorectal TO.

For this purpose, we chose the modified Delphi method, a validated technique already applied to detect the TO for both hepatic and pancreatic surgery with an international expert panel [13,24]. To our knowledge, this is the first Italian study on the textbook outcome in colorectal surgery. The national expert panel was established after the meticulous identification of high-expertise surgeons in both colorectal oncology surgery and academic research.

The respondents assessed a total of 26 questions in 5 surgical domains using a modified two-round Delphi process and defined the colorectal TO as the presence of 90-day postoperative survival, negative margins (5 cm for colon resections) and at least 12 lymph nodes, a minimally invasive approach if available and not contraindicated, ostomy fashioning if preoperatively planned (only for colon resections), postoperative complication of a grade less than Clavien–Dindo 3b, at least 10 ERAS items if applicable, no unscheduled readmission (within 30 days), proper CHT and RT regimens, a complete colonoscopy after or before surgery and Tumor Board Evaluation.

The first topic of the current study was postoperative survival, and it was the most debated statement. After two voting rounds, 90-day survival was lastly chosen by the panelists as the appropriate time frame to perform survival analysis. This definition is in line with the literature. A large retrospective study analyzed 171,688 patients submitted to colorectal resections for cancer with the aim of defining how mortality outlier status at 90 days compares with death at 30 days. The authors concluded that the 90-day death rate is a better indicator of perioperative outcomes as compared with 30-day mortality, allowing the inclusion of a greater number of mortality outliers [25]. Furthermore, in 2018, Adam et al. reported that 90-day survival is a better measure of hospital performance in oncologic colorectal surgery in comparison with 30-day survival [26].

It is not clear why 90-day survival leads to the identification of a greater number of mortality outliers than at 30 days. One reason could be that this indicator also gathers information on postdischarge deaths, with or without readmission, due to severe postdischarge complications, inadequate deep vein thrombosis prophylaxis, postdischarge variation in failure-to-rescue cases (rate of patients deceased after diagnosis of a complication) and patient follow-up practices. This outcome may partially reflects hospitals’ competences in managing complications; furthermore, it can also be affected by other system deficiencies such as critical care and radiology performance [25].

Moreover, some factors influenced delayed mortality, such as being elderly, male gender, a lack of insurance, comorbidities, higher tumor grade and stages, more extensive resections and treatment at low-volume centers [26].

The second item was radical resection, which is the main parameter used to establish a correct oncologic resection. It was defined as an adequate number of lymph nodes yielded and proper resection margins. Regarding R0 resection, the consensus defined an adequate number of lymph nodes (≥12) as a criterion for oncological radicality, and a 5 cm free resection margin was confirmed only for colic resections.

This statement is in line with the main guidelines [3,4,5]; indeed, the most recent ESMO guideline reported the following: “The resection should include a segment of colon of at least 5 cm on either side of the tumour…at least 12 lymph nodes should be resected when feasible” [5].

The MI approach was found to be predictive of achieving the TO in the absence of contraindications. MI surgery may be considered based on the following well-defined criteria: high surgeon experience and skill, usually is not suggested for locally advanced tumor or complicated cancer (obstruction and/or perforation) and the lack of a need for thorough abdominal exploration [4,5].

In the last two decades, improvements in the surgical management of CRC have drastically developed into a more MI approach with unequivocal benefits in terms of postoperative outcomes [27,28].

Another debated concern was about ostomy fashioning. After discussion, the respondents concluded that ostomy cannot be a valid reason to exclude a patient from a TO; in fact, often, it does not correspond to an irregular operative course but to a preoperative plan. The panelists concluded that the TO can be evaluated according to the preoperative decisions: if an ostomy was planned before surgery, it still can be included in the textbook outcome.

To the best of our knowledge, some patients with CRC required planned stoma formation; as a matter of fact, the ESMO guidelines state that “Obstructive CRCs can be managed in one or two stages. Two-stage procedures can include colostomy followed by colonic resection or, in the case of bowel perforation, Hartmann’s procedure followed by colostomy closure and anastomosis”. Otherwise, several authors recommend constructing a protective stoma to avoid severe grades of leakage in high-risk patients that could lead them to develop anastomotic dehiscence [6,29]. The respondents’ decision regarding stoma formation is consistent with the colorectal TO proposed by Manatakis et al., whilst Maeda et al. excluded ostomy fashioning from the TO definition, underlining that this is also a debated issue in other colorectal TOs. [16,30].

It is noteworthy that the panelists did not reach consensus for the maximal LOS, even though it is often used as a main indicator of postoperative course quality, especially in Italy, where the median LOS is one of the criteria adopted by national agencies to determine surgical units’ quality. The absence of LOS in this version of the TO is consistent with the TOs of others specialties [13,31,32]. A prolonged LOS is not always associated with a complicated course and slow recovery, but also depends on cultural differences, recovery protocols (ERAS), prehabilitation programs and both the availability and accessibility of post-acute care institutions [22,33,34].

The respondents identified as a TO item the necessity to carry out a complete endoscopy after or before surgery, because colonoscopy allows us to precisely locate, mark and biopsy the cancer. Furthermore, it enables us to identify and remove synchronous precancerous or cancerous lesions. The ESMO guidelines strongly recommend that “in cases where complete colonic exploration cannot be carried out before surgery, a complete colonoscopy should be carried out within 3–6 months” [5]. Consistent with the data from the literature, the number of patients that reach the TO for colon cancer surgery is no more than 60% [11,35]. The TO percentages are slightly higher for low-risk patients, who are more able to make informed decisions about hospital choice, travel for better quality of treatment and are mostly healed in an elective context. As a matter of fact, the TO does not take into consideration patients’ nonmodifiable characteristics; indeed, the following is the “ideal” perioperative course of a perfect patient: low BMI, ASA ≤ II, young, without significant comorbidity, without previous abdominal surgery and with an early tumor. Hence, there is no standardization in our surgical operation so it is very difficult to ask for a TO that is based on standardized variables. Another way to try to check the quality of care provided by hospitals, departments and individual surgeons is using a benchmark. A benchmark means that the best patient can be chosen, and for this patient, the perioperative course must be perfect. A benchmark describes a “best possible” outcome under ideal conditions. This tool could overcome the clinical–epidemiological differences that occur in real life. Unfortunately, the benchmark patient represents only 1 in 10 in Western countries, while in Eastern countries, they represent 1 in 4 [36]. Therefore, in real life, it is difficult to set a standard, especially for non-benchmark patients.

The strong points of our study are the elevated expertise of the selected panel in both surgical activity and academic research, the anonymity during all the Delphi stages, the anonymity of the panelists, responses and comments, and the presence of controlled feedback during all the voting phases.

The current study has many limitations.

First, it includes both colon and rectal cancers, which are two different entities in terms of diagnosis, treatment and postoperative courses.

Second, this type of Delphi consensus lacked potentially important perspectives of clinicians from other disciplines involved in patients’ multidisciplinary management.

Third, the expert panel was composed mainly of males, with only 10.7% being women. Even though several improvements have been made, the current situation of women in surgery is still characterized by career barriers, with a worldwide underrepresentation of female surgeons in leadership positions and senior academic rankings [37,38]. Further positive steps are needed to achieve gender equity and inclusion in the surgical field.

We expect that this Italian version of the colorectal TO will be disseminated among major colorectal cancer surgery centers with the aim of identifying potential weaknesses and strengths and increasing the surgical quality offered to the patient.

## 5. Conclusions

To our knowledge, this is the first Italian study on the textbook outcome in colorectal surgery. This Italian TO was defined as the presence of 90-day postoperative survival, negative margins and at least 12 lymph nodes, a minimally invasive approach, ostomy fashioning if preoperatively planned, a postoperative complication < Clavien–Dindo 3b, at least 10 ERAS items, no readmission, proper CHT and RT regimens, complete colonoscopy after or before surgery and Tumor Board Evaluation. The version we propose can be used to verify adherence to all items of the textbook outcome. Further studies are needed to validate the application of the TO in daily clinical practice and to evaluate the use of this composite tool in surgical quality assessment.

## Figures and Tables

**Table 1:** Expert panelists’ features and demographics.

		*n*	%
Gender	Male	25	89.3
	Female	3	10.7
Hospital setting	Teaching	27	96.4
	Non-teaching	1	3.6
Colorectal surgery expertise	<5 y	1	3.6
	≥5 y <10 y	2	7.1
	≥10 y	25	89.3
Number of colorectal resections	<500	15	53.5
	≥500	13	46.5
Teaching activity	Yes	27	96.4
	No	1	3.6
Speaker, moderator or session president at international and national meetings	Yes	28	100.0
	No	0	0.0
Journal board activity	Yes	23	82.2
	No	5	17.8
Number of scientific publications	<50	8	28.5
	≥50 <100	9	32.2
	≥100	11	39.3
H-index	<10	2	7.2
	≥10 ≤20	5	17.8
	>20	21	75.0

**Table 2:** Results—items that reached consensus.

Item	Median (IQR)	Round of Consensus	Agreement, % (*n*/N)
1.2 30-day survival	4 (3–5)	1	75.0 (21/28)
1.3 90-day survival (no mortality for all causes)	3.5 (3–5)	1	75.0 (21/28)
2.2 Negative margins	4 (3–5)	1	85.7 (24/28)
2.3 ≥12 LNs harvested	4 (3–4.25)	1	78.6 (22/28)
2.5 Negative margins + 12 LNs	4 (3–5)	1	75.0 (21/28)
2.7 Negative margins + 12 LNs + 5 cm for colon	4 (3–5)	1	85.7 (24/28)
3.1 Ostomy fashioning	4 (3–5)	2	92.6 (25/27)
3.2 Minimally invasive approach	4 (3–5)	1	89.3 (25/28)
4.1 No reinterventions	4 (3–5)	1	82.1 (23/28)
4.2 No complications	4 (3–5)	1	78.6 (22/28)
4.6 No readmission (30 days)	4 (3–5)	1	85.7 (24/28)
4.8 ERAS, at least 10 items	4 (3–5)	1	75.0 (21/28)
4.9 ERAS, according to comorbidities of the patient	3 (2.75–5)	1	75.0 (21/28)
4.9 Clavien–Dindo grade < 3b	4 (4–5)	2	88.9 (24/27)
5.1 Appropriate CHT and RT regimens	3 (2.75–4)	1	75.0 (21/28)
5.2 CHT and RT for rectal cancer	4 (4–5)	1	92.9 (26/28)
5.3 Complete colonoscopy after or before surgery	4.5 (2.75–5)	1	75.0 (21/28)
5.4 Tumor Board Evaluation	5 (4–5)	1	96.4 (21/28)

IQR: interquartile range; LN: lymph node; ERAS: Enhanced Recovery After Surgery; CHT: chemotherapy; RT: radiotherapy; *n*: number of agree responses; N: total responses.

**Table 3:** Results—items that did not reach consensus.

Item	Round 1		Round 2	
	Median (IQR)	Agreement, % (*n*/N)	Median (IQR)	Agreement, % (*n*/N)
1.1 Survival until discharge	3 (1–4)	53.6 (15/28)		
1.4 90 d survival (surg. causes)	2.5 (2–4)	50.0 (14/28)	3 (1–5)	59.3 (16/27)
2.1 Radical resection	1 (1–2)	17.9 (5/28)		
2.4 No margins of 5 cm (only for colon)	3 (2–3.25)	60.7 (17/28)	4 (2–5)	66.7 (18/27)
2.6 Negative margins + 5 cm for colon	3 (2–4)	60.7 (17/28)		
4.3 LOS < 7 days	3 (2–4)	57.1 (16/28)	3 (2.5–4)	74.1 (20/27)
4.4 LOS < 75 perc.	3 (2–4)	64.3 (18/28)		
4.5 No readmission (90 days)	3 (2–4)	64.3 (18/28)		
4.7 ERAS, all items	3 (2–4)	57.1 (16/28)		

IQR: interquartile range; LOS: length of stay; *n*: number of agree responses; N: total responses; ERAS: Enhanced Recovery After Surgery.

**Table 4:** Textbook outcome, Italian Version.

Textbook Outcome, Italian Version		
Items		Agreement, % (*n*/N)
Postoperative survival	90-day survival	75.0 (21/28)
Resection R0	Negative margins: longitudinal (5 cm for colon) and circumferential	75.0 (21/28)
	≥12 LNs harvested	78.6 (22/28)
Optimal surgery	Minimally invasive approach if available and not contraindicated	89.3 (25/28)
	Ostomy fashioning only if preoperatively planned	92.6 (25/27)
Regular postoperative course	Clavien–Dindo < 3b	88.9 (24/27)
	ERAS with at least 10 items if applicable	77.8 (21/27)
	No unscheduled readmission (within 30 days)	85.7 (24/28)
Adequate perioperative treatments and diagnostics	Appropriate CHT and RT regimens	75.0 (21/28)
	Complete colonoscopy after or before surgery	75.0 (21/28)
	Tumor Board Evaluation	96.4 (21/28)

LN: lymph node; ERAS: Enhanced Recovery After Surgery; CHT: chemotherapy; RT: radiotherapy; *n*: number of agree responses; N: total responses.

## Data Availability

The original contributions presented in the study are included in the article/Supplementary Materials, further inquiries can be directed to the corresponding author.

## Supplementary Materials

The following supporting information can be downloaded at: https://www.mdpi.com/article/10.3390/jcm13164687/s1, File S1: Delphi rounds 1 and 2.
