# Supplementary material for: Textbook Outcome in Colorectal Surgery for Cancer: An Italian Version
Source: J Clin Med. 2024 Aug 9;13(16):4687. doi: 10.3390/jcm13164687 (PMC11355911; doi:10.3390/jcm13164687)

Textbook Outcome in Colorectal surgery for cancer: an Italian version
Supplementary material

Round 1

Statement 1: Survival
In a textbook outcome in colorectal surgery for cancer, survival outcome must be described as:
1.1 hospital survival, defined as survival during all postoperative course, from surgery until discharge to home
1.2 30-day survival, defined as survival until 30 days after surgery
1.3 90-day all cause-mortality, defined as survival until 90 days after surgery taking in account mortality for all causes and not only directly connected to surgery
1.4 90-day taking in account mortality only directly connected to surgery

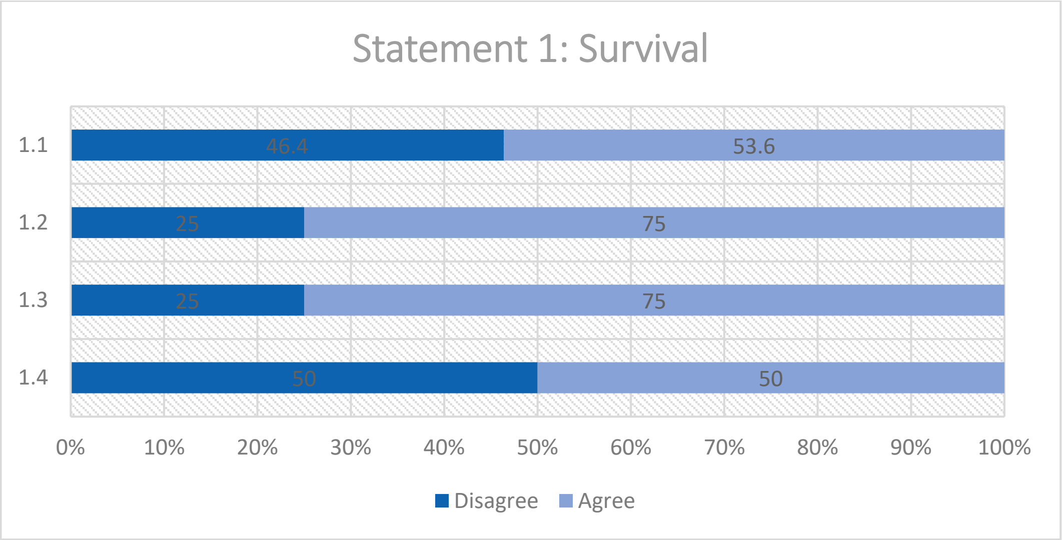

Statement 2: Oncologic Radicality obtained during colorectal surgery should be expressed as:
2.1 A general definition of “radical resection”
2.2 Negative microscopic margins (R0), defined as resection to negative margins: proximal, distal and circumferential (CRM)
2.3 An AJCC compliant lymph-node count should be considered too, with more than 12 lymph-nodes harvested
2.4 Proximal and distal resection margins length at least 5 cm (ONLY FOR COLON CANCER)
2.5 2.2 + 2.3 together
2.6 2.2 + 2.4 together
2.7 2.2 + 2.3 + 2.4 together

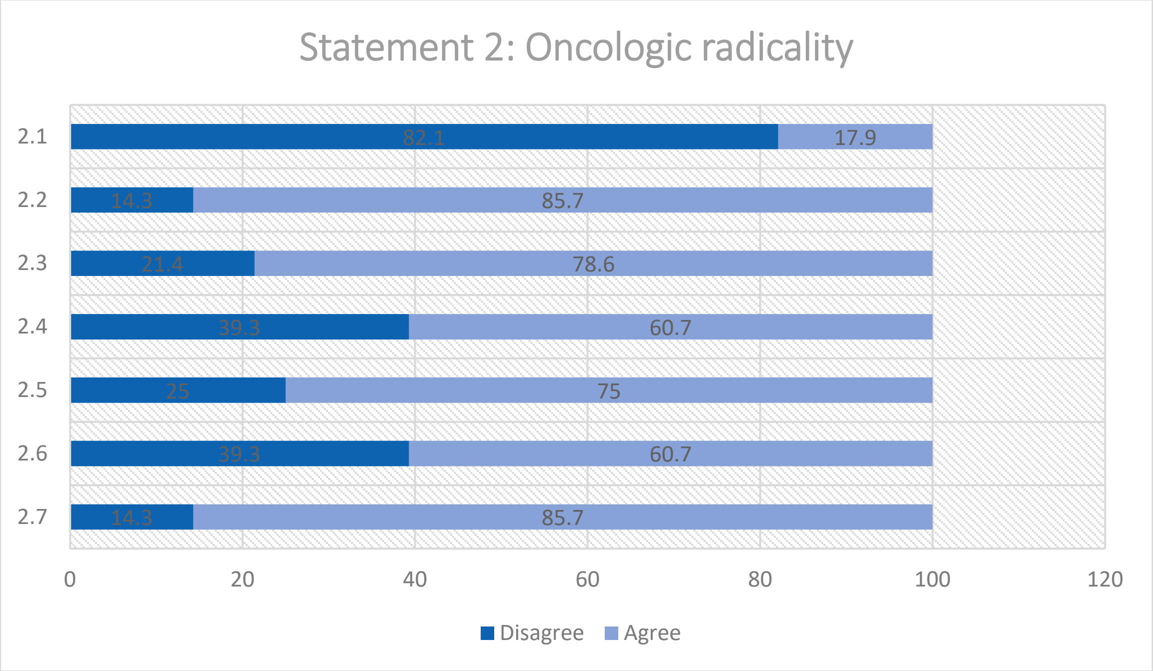

Statement 3: Surgery.
3.1 ONLY FOR COLON CANCER: With regard to the colonic surgery, lack of ostomy should be excluded from items of textbook outcome
3.2 Minimally invasive approach unless contraindicated

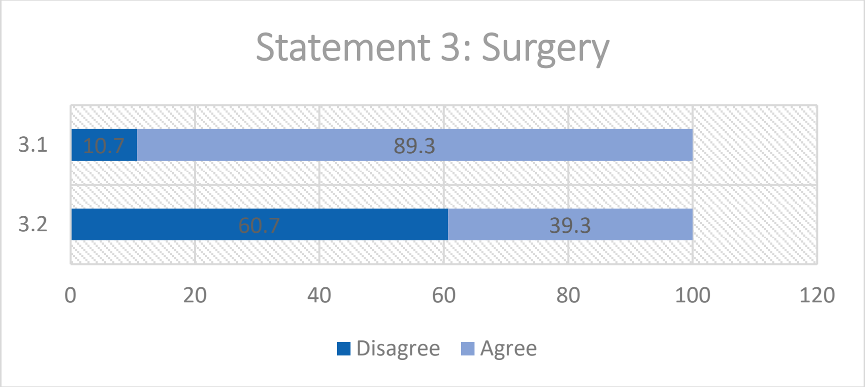

Statement 4: Post-operative course
Post-operative outcomes in a textbook colorectal surgery must include:
4.1 no reintervention in the postoperative course
4.2 no adverse outcomes
4.3 hospital stay of 7 days or less
4.4 no prolonged postoperative length of stay (LOS) defined as ≤ 75th percentile by year
4.5 no readmission during 90 days after discharge
4.6 no unplanned 30-day readmission
4.7 employment of ERAS protocol (all items)
4.8 employment of ERAS protocol (at least 10 items)
4.9 employment of ERAS protocol according to general conditions and comorbidities of the patient

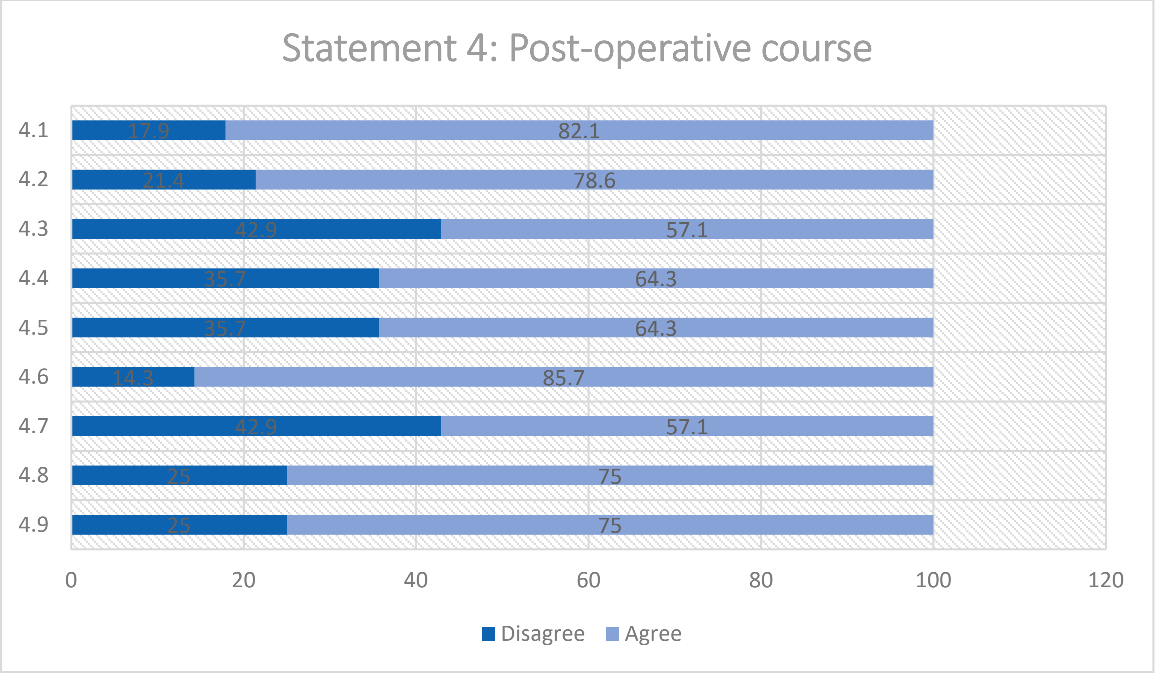

Statement 5: An adequate chemo-radiation treatment of patients with colorectal cancer should include:

5.1 Appropriate chemotherapy starting within 4 months after diagnosis for patients < 80 years old with pathologic stage 3 disease b,c

5.2 ONLY FOR RECTAL CANCER: Receipt of neoadjuvant multi-agent chemo and radiotherapy, total neoadjuvant therapy (induction chemotherapy followed by chemo-radiotherapy) or both neoadjuvant and adjuvant chemo-radiotherapy for clinically locally advanced (cT3–T4b and/or cN1–N2b) disease

5.3 A complete colonoscopy before or after surgery within 6 months

5.4 Tumor board evaluation to discuss the management of the patient with colorectal cancer

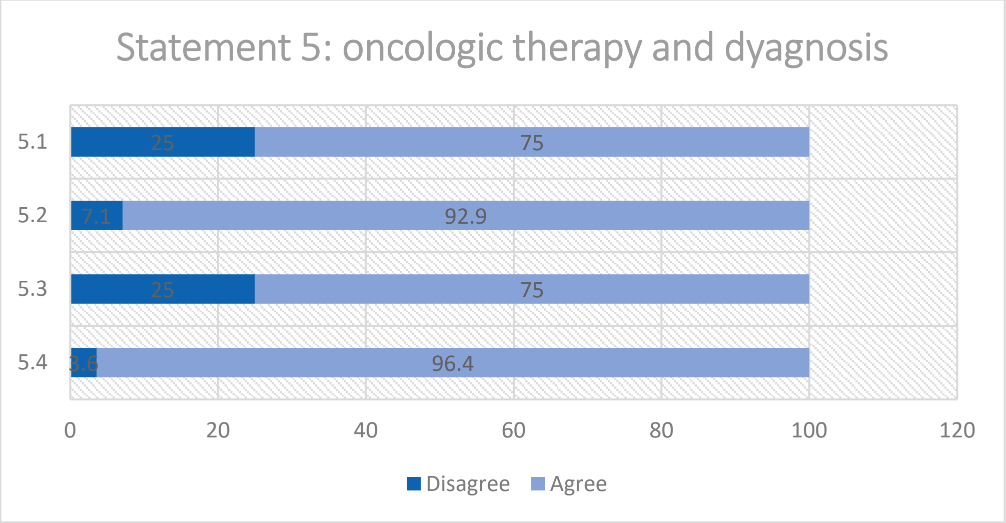

Round 2

Statement 1: Survival
In a textbook outcome in colorectal surgery for cancer, survival outcome must be described as:
1.3 mortality within 90 days due to any causes
1.4 mortality within 90 days only related to surgery

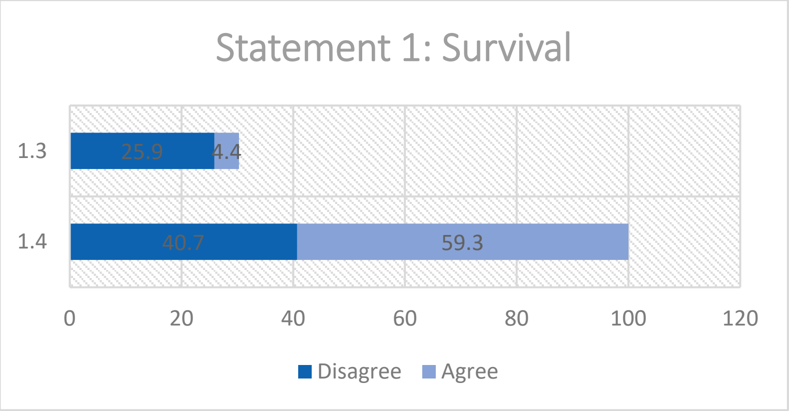

Statement 2: Oncologic Radicality obtained during colorectal surgery should be expressed as:
2.3 Oncological radicality is achieved by negative margins OR by Lymph nodes yield ≥ 12
2.4 Oncological radicality is achieved by negative margins AND by Lymph nodes yield ≥ 12
2.5 Resection margins length of 5 cm has to be removed from the definition of oncological radicality in a textbook outcome

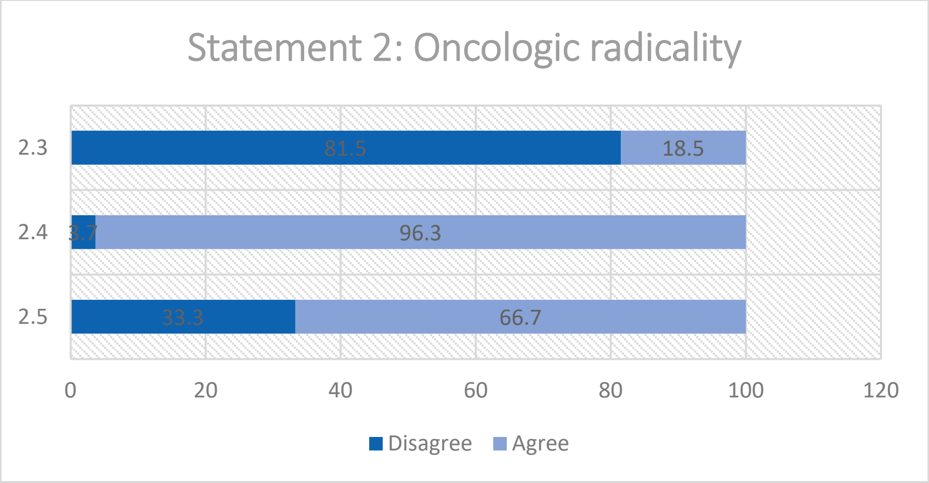

Statement 3: Surgery.
3.1 Ostomy can be considered a deviation from surgical optimal course only if fashioned for intra- or post-operative complications

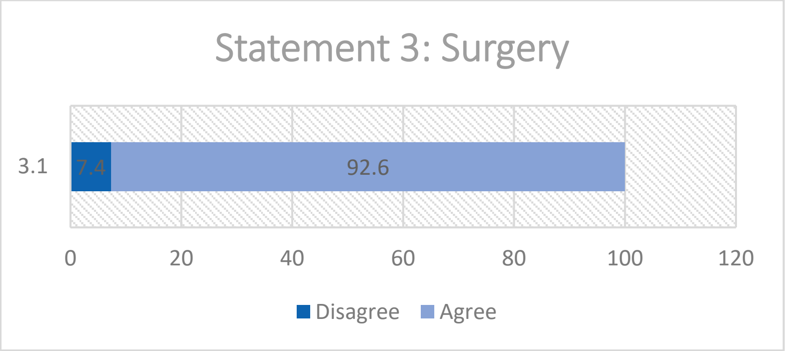

Statement 4: Post-operative course
Post-operative outcomes in a textbook colorectal surgery must include:
4.3 Length of hospital stay of 7 days or less is part of an optimal postoperative course after COLON surgery
4.4 Length of hospital stay of 7 days or less is part of an optimal postoperative course after RECTAL surgery
4.5 Length of hospital stay of 10 days or less is part of an optimal postoperative course after RECTAL surgery
4.7 ERAS protocol should be implemented to fulfill at least 10 items
4.10 An optimal postoperative course should not have ≥ 3b Clavien-Dindo complication

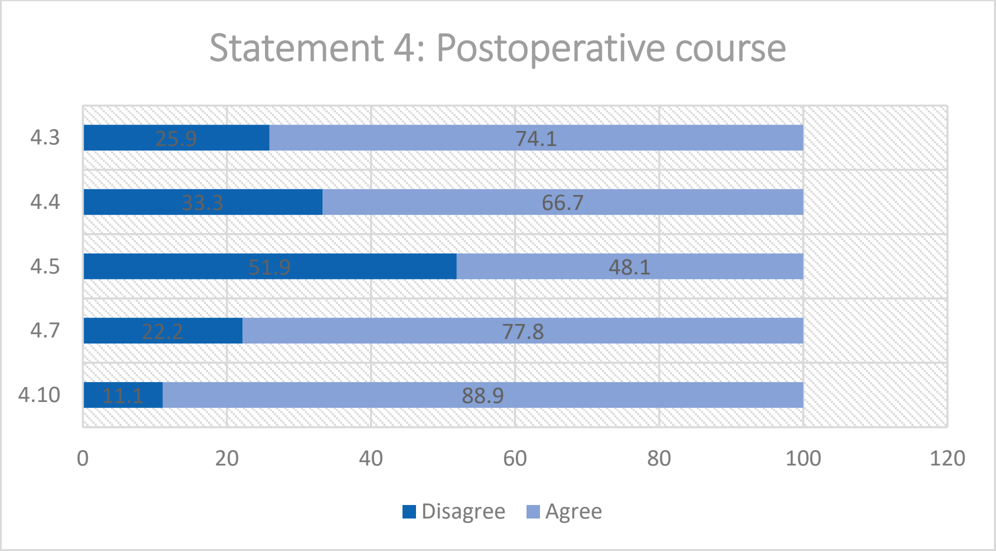

Supplement: Supplementary file 1 [file jcm-13-04687-s001.zip › jcm-3084469-supplementary.pdf]
